# Efficacy, tolerability, and safety of aripiprazole once-monthly versus other long-acting injectable antipsychotic therapies in the maintenance treatment of schizophrenia: a mixed treatment comparison of double-blind randomized clinical trials

**DOI:** 10.3402/jmahp.v3.27208

**Published:** 2015-09-10

**Authors:** Istvan M. Majer, Fiona Gaughran, Christophe Sapin, Maud Beillat, Maarten Treur

**Affiliations:** 1 Pharmerit International, Rotterdam, The Netherlands; 2 National Psychosis Service, South London and Maudsley NHS Foundation Trust, London, United Kingdom; 3 The Institute of Psychiatry, Psychology and Neuroscience, King's College, London, United Kingdom; 4 Global Analytics, Lundbeck SAS, Paris, France; 5 Global Health Economics & Outcomes Research, Lundbeck SAS, Paris, France

**Keywords:** efficacy, long-acting injectable antipsychotics, maintenance treatment, mixed treatment comparison, safety, schizophrenia

## Abstract

**Background:**

Treatment with long-acting injectable (LAI) antipsychotic medication is an important element of relapse prevention in schizophrenia. Recently, the intramuscular once-monthly formulation of aripiprazole received marketing approval in Europe and the United States for schizophrenia.

**Objective:**

This study aimed to compare aripiprazole once-monthly with other LAI antipsychotics in terms of efficacy, tolerability, and safety.

**Data sources:**

A systematic literature review was conducted to identify relevant double-blind randomized clinical trials of LAIs conducted in the maintenance treatment of schizophrenia. MEDLINE, MEDLINE In-Process, Embase, the Cochrane Library, PsycINFO, conference proceedings, clinical trial registries, and the reference lists of key review articles were searched. The literature search covered studies dating from January 2002 to May 2013.

**Study selection:**

Studies were required to have ≥24 weeks of follow-up. Patients had to be stable at randomization. Studies were not eligible for inclusion if efficacy of acute and maintenance phase treatment was not reported separately. Six trials were identified (0.5% of initially identified studies), allowing comparisons of aripiprazole once-monthly, risperidone LAI, paliperidone palmitate, olanzapine pamoate, haloperidol depot, and placebo.

**Data extraction:**

Data extracted included study details, study duration, the total number of patients in each treatment arm, efficacy, tolerability, and safety outcomes. The efficacy outcome contained the number of patients that experienced a relapse, tolerability outcomes included the number of patients that discontinued treatment due to treatment-related adverse events (AEs), and that discontinued treatment due to reasons other than AEs (e.g., loss to follow-up). Safety outcomes included the incidence of clinically relevant weight gain and extrapyramidal symptoms.

**Data synthesis:**

Data were analyzed by applying a mixed treatment comparison competing risks model (efficacy) and using binary models (safety). There was no statistically significant difference between any study outcome, including the risk of relapse, the risk of discontinuations, and safety outcomes.

**Conclusions:**

Aripiprazole once-monthly is similarly efficacious to other LAIs with relatively low rates of discontinuation due to AEs and due to reasons other than AEs than other LAIs.

Schizophrenia is a chronic mental disorder characterized by positive symptoms (e.g., hallucinations, delusions, and behavioral disturbances), which may clear to reveal negative symptoms (e.g., lack of emotion, reduced interest in day-to-day activities, social withdrawal, poor general psychopathology, and functioning) and cognitive impairment. The course of schizophrenia often includes acute exacerbations or ‘relapses’, which necessitate additional medical care (1). The symptoms of schizophrenia can worsen with repeated relapses causing social functioning of the patients to deteriorate over time (2).

Overall, schizophrenia has a substantial economic impact on the patient and society as a whole. While the lifetime prevalence and incidence are 0.3–0.7% and 10–22 per 100,000 person-years, respectively (3, 4), its management accounts for an estimated 1.5–3% of total national healthcare expenditures (5). Costs related to relapse are particularly high, constituting ≥60% of all direct medical costs (1). To reduce the economic and humanistic burden of schizophrenia, maintenance treatment with antipsychotic medication has become a fundamental element of disease management and is supported by available treatment guidelines (6, 7).

Although long-term and continuous intake of medication as prescribed is essential for effective maintenance treatment, non-adherence to oral antipsychotics is a recognized and major problem in a significant proportion of patients. Numerous studies have documented the rates of non-adherence; the latest systematic reviews found that approximately 50% of patients are partially or totally non-adherent with oral antipsychotics (8, 9). The short-term and long-term consequences of non-adherence include symptom exacerbation, elevated risk of relapse, and increased utilization of healthcare resources (10–13). In a recent 3-year follow-up study, non-adherence was identified as the most important predictor of relapse (11). Furthermore, non-adherence makes it difficult for psychiatrists to assess treatment response and to make appropriate adjustments to therapy (9, 14).

To promote adherence to treatment regimens, long-acting injectable (LAI) formulations have been developed as an alternative to oral antipsychotics. LAIs are administered by intramuscular injection and a single injection lasts in the range of 1 week to 1 month, depending on compound and formulation (15). LAIs relieve patients from the daily need to take medication and subsequently have the potential to improve adherence. In addition, failure to attend injection visits can serve as a warning sign for the physician for non-adherence. Ultimately, LAIs have been suggested to result in improved clinical and economic outcomes (16, 17). In the past, LAIs were primarily recommended for treating patients who were non-compliant with oral medication, whereas latterly LAIs are also prescribed for those who choose to receive LAI antipsychotics. Recently, it has been suggested that the use of LAIs may lead to the most health gains in early episode patients for whom schizophrenia is most treatable (18).

Before 2013, there were six first-generation LAI antipsychotics (FGA), that is, flupentixol, fluphenazine, haloperidol, perphenazine, pipotiazine, and zuclopenthixol, and three second-generation LAI antipsychotics (SGA), that is, risperidone LAI, olanzapine pamoate, and paliperidone palmitate, available for patients in Europe. In general, FGAs are associated with a different side effects profile than SGAs with a higher risk of movement disorders, for example, extrapyramidal symptoms (EPS) and tardive dyskinesia, although SGAs have been more linked to weight gain and metabolic risk (15). Compared to FGAs, SGAs had been widely believed to represent an advance in the long-term management of the illness however in pragmatic trials SGAs were not found to be more effective than FGAs (19).

Recently, the intramuscular once-monthly (OM) formulation of aripiprazole received positive opinion from the Committee for Medicinal Products for Human Use in the European Union (20) for the maintenance treatment of schizophrenia and has been approved by the Food and Drug Administration (FDA) in the United States (21) for the treatment of schizophrenia.

No head-to-head trials have been conducted that compared aripiprazole OM against another LAI antipsychotic medication in the maintenance treatment of schizophrenia. Therefore, the comparative clinical evidence of aripiprazole OM versus comparators could only be generated indirectly. In the past, several meta-analyses have been conducted that studied the psychopharmacological profile of antipsychotics as a whole, that is, numerous treatments combined (22–26); however, to our knowledge, this is the first formal analysis that compared the efficacy and safety profile of individual LAI antipsychotics against each other and placebo. Therefore, the aim of the presented research was to generate estimates of relative efficacy, tolerability, and safety for first- and second-generation LAI antipsychotic treatments using available evidence.

## Methods

### Systematic literature review

#### Study selection

A systematic literature review was conducted to identify studies comparing the efficacy and tolerability of antipsychotic medications in the maintenance treatment of schizophrenia. The search protocol was based on the strategy outlined in the guideline on core interventions in the treatment and management of schizophrenia, issued by the National Institute on Clinical and Health Excellence (NICE) in 2010 (27) and in the updated version published in 2014 (28). The NICE literature review considered randomized controlled trials (RCTs) covering oral and depot as well as first-generation or second-generation antipsychotic therapies administered in maintenance treatment. Blinding of RCTs was restricted to double-blind designs. Studies dating from 1 January 2002 to 30 July 2008 were covered and were included if they had ≥10 adult participants per treatment arm and had long-term follow-up (24 weeks or more). In the NICE literature review, a total of 17 RCTs (*N*=3,535) met the inclusion criteria. The RCTs compared eight different oral antipsychotics with placebo or active treatment. Follow-up time of the RCTs ranged from 26 to 104 weeks.

To update the evidence with recent RCTs, the search period defined in the NICE review was extended up to May 2013. The same search strategy was used for the update except that the search was restricted to RCTs that included at least one LAI antipsychotic treatment in the study arms. No further modifications compared to the NICE protocol were made. Interventions of interest included aripiprazole OM, paliperidone palmitate, olanzapine pamoate, risperidone LAI, and haloperidol depot. There was no restriction imposed on the comparator treatments. Studies including patients with treatment resistant schizophrenia and studies with high proportion (i.e., >10%) of patients suffering from schizoaffective disorder were excluded. A complete overview of the study inclusion and exclusion criteria is presented in the Supplementary file.

MEDLINE, MEDLINE In-Process, Embase, the Cochrane Library, and PsycINFO databases were searched for relevant studies. Electronic database searches were supplemented by manual search of conference proceedings, clinical trial registries (www.clinicaltrials.gov), and the reference lists of key review articles. Similarly to the strategy of the NICE literature review, studies with less than 10 participants, with a time horizon of less than 6 months (or 24 weeks), or of non-English language were excluded. Patients had to be 18 years or older. Studies were excluded if they had a broader scope of research but contained no subgroup analysis for these patients.

The literature searches yielded an initial set of records that were assessed for inclusion or exclusion by two independent reviewers (IMM and MJT) based on citation details (e.g., title, keywords, and abstract) supplied with each citation. Those that did not match the eligibility criteria were excluded. Any disagreements between the reviewers were resolved through discussion until a consensus was reached. The categorization process was undertaken using an inclusive approach to ensure records were included rather than excluded if the topic was unclear. Duplicates of citations (due to overlap in the coverage of the databases) were also excluded in the first pass. In instances when it was not possible to include or exclude citations based on the abstract, full-text copies were ordered. Selected articles underwent another round of selection process (full-text review) by two independent reviewers (IMM and MJT). Any disagreements between the reviewers over inclusion or exclusion were resolved by discussion until a consensus was reached.

#### Data extraction

From each included study four types of information were extracted: citation details, study design including study duration, the total number of patients in each study arm, as well as efficacy, tolerability, and safety outcomes. The efficacy and tolerability outcomes included four mutually exclusive events: relapse (including the definition used), treatment discontinuation due to adverse events (AEs), treatment discontinuation due to reasons other than AEs, and continuation of treatment until the end of study follow-up. The safety outcomes included incidence of clinically relevant weight gain (i.e., >7% increase compared to baseline) and incidence of EPS during the maintenance treatment. EPS was defined either by the number of patients using anticholinergic medication (if this information was available) as concomitant medication or by the number of patients experiencing specific symptoms, including tardive dyskinesia, parkinsonism, or akathisia. Data extraction was carried out by two independent reviewers (IMM and MH) who recorded all specified information in a separate data extraction file. In accordance with the Centre for Reviews and Dissemination (CRD) and Cochrane guidelines for quality assessment of RCTs, different types of potential biases (selection bias, performance bias, detection bias, attrition bias, and reporting bias) were assessed (29). Quality assessments were reported using the suggested format by NICE (30).

### Data synthesis

**Fig. 1:**
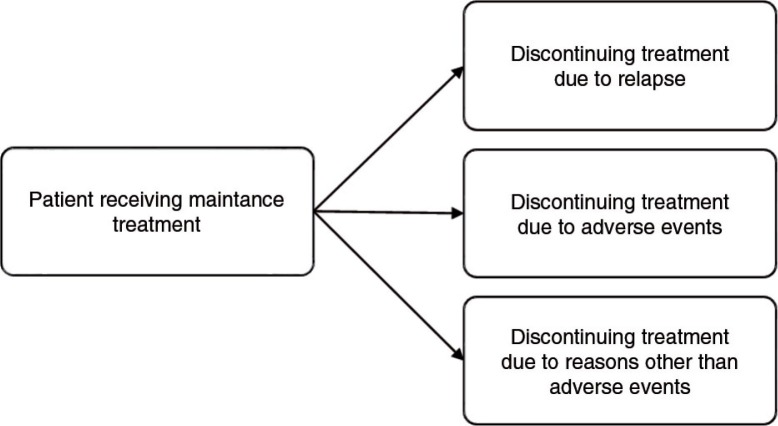
Competing events structure of the mixed treatment comparison.

A mixed treatment comparison (MTC) model was set up to jointly analyze the efficacy and tolerability outcomes extracted from the relevant RCTs identified by the literature review (31). Since the outcomes (i.e., relapse, discontinuation due to treatment-related AEs, discontinuation due to reasons other than AEs, and continuation of treatment) were mutually exclusive, that is only one of the events could occur for a patient during the study follow-up, a competing risk model was used. In essence, the model for the efficacy and tolerability analysis was identical to previously proposed MTC models for binomial data, except that a multinomial likelihood and linking function were used, as is appropriate for the evidence structure at hand (32). Figure 1 presents the competing events structure of the MTC. Separate MTCs with binary data were used for the safety outcomes (i.e., clinically relevant weight gain and EPS).

The MTC models were constructed following the principles of Bayesian analysis (33). Uninformative priors were imposed to prevent making assumptions on the model outcomes, which was in accordance with the methodological recommendations made by NICE on MTCs of RCTs. Goodness of fit was tested using the deviance information criterion (DIC) tool. For each MTC analysis two different models were tested: a fixed effects model and a random effects model allowing between-trials variance to vary by outcome. The DIC was used to determine which model fitted the data better. Convergence was assessed by inspection of the Gelman–Rubin statistics (34), time series plots of the sample estimates, and autocorrelation plots.

For the efficacy and tolerability analysis the hazard ratio (HR) of relapse, the HR of discontinuation due to AEs, and the HR of discontinuation due to other reasons were estimated simultaneously for each active treatment relative to placebo. Probabilities of relapse, treatment discontinuation due to AEs, and discontinuation due to other reasons over 26 weeks (i.e., 6 months) for each therapeutic intervention of interest were estimated as well; the probability of continuing therapy was determined by one minus the sum of the three discontinuation probabilities. For the safety outcomes, odds ratios of clinically relevant weight gain, and EPS for the active treatments relative to placebo were estimated. Using the estimated baseline probability and odds ratios, the incidence risk of these AEs was calculated. In each analysis, placebo (depot) was chosen as the reference treatment for ease of comparability and interpretability of the results. To examine the robustness of the model results, various different sensitivity analyses were performed. These included an analysis of the fixed effects model, an analysis suggested by the Decision Support Unit (DSU) (35) of NICE assuming zero between-trial correlation for the efficacy endpoints, an analysis that included only studies which used true placebo depot, an analysis that excluded risperidone LAI and haloperidol depot from the evidence network, separately, and an analysis that assumed different durations for the placebo-controlled aripiprazole OM trial terminated early (i.e., time horizon 44 weeks instead of 52 weeks).

The models were conducted using Markov Chain Monte Carlo (MCMC) simulation methods implemented in WinBUGS 1.4. In total, 360,000 iterations were run, after which the first 60,000 iterations were discarded. Every 30th simulation was retained to ensure independence between the simulations. Accordingly, 10,000 posterior simulations were recorded. The mean of the posterior distribution was taken as the point estimate, and 95% credible intervals (CrIs) were calculated as well. In addition, the probability of each treatment being the best in ranking with respect to each of the outcomes was reported for the efficacy and tolerability model as well as for the safety models. Details of the adapted methodology can be found in the Supplementary file, in the study of Ades et al. (32), or in the NICE guideline on core interventions in the treatment and management of schizophrenia (27).

## Results

### Systematic literature review

After removing duplicates from search results of different electronic databases, 95 studies were selected for full-text screening, of which 16 studies presented data with outcomes of interest in the maintenance treatment. Six RCTs assessing depot LAIs were identified eligible for inclusion (see Supplementary file for the PRISMA-diagram) (36). Studies were excluded mainly for not reporting efficacy and tolerability data for the predefined endpoints, for including patients with other diseases than schizophrenia, or for inappropriate study design (e.g., open-label design during the maintenance phase, short duration of trial, acute treatment setting). While most RCTs were excluded from the evidence base, the application of strict selection criteria was essential for identifying studies with homogeneous patient populations.

Among these six RCTs, one study (37) compared the efficacy and safety of paliperidone palmitate versus risperidone LAI in a slightly different population (i.e., patients with acute disease). However, it was considered a relevant addition to the final set of RCTs for a number of reasons. First, it included risperidone LAI, which was an essential piece for the evidence base. Second, it had a double-blinded, randomized trial design with long period of follow-up that allowed the extraction of sufficiently mature data. Third, all outcomes of interest were reported for the combined acute and maintenance phase.

Ultimately, six RCTs were included for the efficacy and safety MTCs, respectively (37–42). These studies formed the evidence base (or evidence network) that allowed comparisons of aripiprazole OM, risperidone LAI, paliperidone palmitate, olanzapine pamoate, haloperidol depot, and placebo depot for the efficacy and tolerability analyses. Two studies considered oral forms of aripiprazole and olanzapine as active references; they are included to ensure full data presentation. Safety data were not available for haloperidol depot in the trial; thus, comparison with haloperidol depot was not feasible for the clinically relevant weight gain and EPS outcomes. The patient populations across the RCTs were homogeneous: average age of the patients was approximately 38–40 years, average disease duration was 13–17 years, and patients were stable. The number of patients included in the studies amounted to 3,394 of which 16% received aripiprazole OM, 19% placebo depot, 2% haloperidol depot, 18% olanzapine pamoate, 17% paliperidone palmitate, 11% risperidone LAI, and 17% oral antipsychotics. Table 1 presents detailed information on the individual study designs and patient characteristics.

**Table 1:** Details of included studies assessing long-acting injectable antipsychotics

Study	Patient characteristics	Inclusion criteria^a^	Exclusion criteria	Definition of relapse-related outcome	Medication	Notes
Kane (42)	Age: ~38–40 Male: 85% White: 59% BMI: not reported Duration (year): 12–17 Schizoaffective: 8 (7.6%) Outpatients only Total PANSS score at baseline: not reported *N*=105; 52 weeks	DSM-III schizophrenia or schizoaffective disorder for at least 2 years; need for maintenance AP treatment; baseline state of relative remission for ≥3 months during maintenance treatment	Treatment with lithium or antidepressants; women of childbearing potential; contraindication of haloperidol; BPRS ≥ 4 for conceptual disorganization/unusual thoughts or BPRS ≥ 5 for hallucinatory behavior/suspiciousness	Symptomatic exacerbation: an increase on one psychotic item of at least 2 scale points on one of the four items of the BPRS	Haloperidol depot: 200 mg/month; 100 mg/month; 50 mg/month (doses were combined for the MTC) Haloperidol depot: 25 mg/month (assumed equivalent to placebo)	Discontinuation due to other reasons than AEs included study termination, patient uncooperative and other
Kane (41)	Age: ~38–39 Male: 67% White: 72% BMI: 26.9 kg/m^2^ Duration (year): 12–14 Schizoaffective: – Outpatients only Total PANSS score at baseline: ~55–58 *N*=1,065 24 weeks	DSM-IV schizophrenia Stability criteria: no dose change for oral olanzapine; CGI-I score ≤4; outpatient for ≥4 weeks; BPRS positive symptom subscale score <5 on conceptual disorganization, unusual thoughts, hallucinatory behavior and suspiciousness	Significant suicidal or homicidal risk; pregnancy/breastfeeding; acute/serious/unstable medical conditions; substance dependence within the past month	Psychotic exacerbation: an increase of any BPRS positive symptom item to a score >4, with an absolute increase ≥2 for the specific item or ≥4 on the positive symptom subscale since randomization; hospitalization as the result of worsening of positive psychotic symptoms	Olanzapine pamoate: 150 mg/2–weeks; 405 mg/4-weeks; 300 mg/2-weeks (doses were combined for the MTC) Olanzapine oral: 10/15/20 mg/day Olanzapine pamoate: 45 mg/4-weeks (assumed equivalent to placebo)	Discontinuation due to other reasons than AEs included loss to follow-up, protocol violation, lack of efficacy (not meeting the definition of relapse), physician decision, patient decision, and sponsor decision
Hough (38)	Age: ~39–40 Male: 55% White: 66% BMI: 27.2 kg/m^2^ Duration (year): 12–13 Schizoaffective: – Outpatients only Total PANSS score at baseline: ~52–53 *N*=410 31 weeks for paliperidone palmitate 42 weeks for placebo depot	DSM-IV schizophrenia for ≥1 year before screening; PANSS score below 120 at screening. Stability criteria: PANSS total score ≤75; PANSS item scores ≤4 on delusions, conceptual disorganization, hallucinatory behavior, suspiciousness, hostility, uncooperativeness, and poor impulse control	Diagnosis other than schizophrenia; significant suicidal or aggressive behavior; substance dependence within the past months; significant medical conditions/treatment resistance; use of 4-week depot within 28 days or RLAI within 5 weeks before screening/oral AP within 2 days; admission to psychiatric hospital	Hospitalization for symptoms of schizophrenia; 25% increase in PANSS total score for two consecutive assessments for patients who scored >40 at randomization, or a 10-point increase for patients who scored ≤40 at randomization; self-injury, suicidal or homicidal ideation and aggressive behavior	Paliperidone palmitate: 25/50/100 mg/month Placebo depot	Time-to-relapse data were censored beyond day 220 for the paliperidone palmitate group (i.e., 220/731 weeks) and beyond day 295 for the placebo group (i.e., 295/742 weeks). Discontinuation due to other reasons than AEs included subject choice, not received any double-blind injections and other
Kane (40)	Age: ~40–42 Male: 60% White: 61% BMI: 28.6 kg/m^2^ Duration (year): 14–15 Schizoaffective: – Outpatients only Total PANSS score at baseline: ~54–55 *N*=403 52 weeks (may be shorter)	DSM-IV-TR diagnosis of schizophrenia for ≥3 years; history of symptom exacerbation or relapse when not receiving AP treatment. Stability criteria: outpatient status; PANSS total score ≤80; PANSS score of ≤4 on conceptual disorganization, hallucinatory behavior and unusual thought content; CGI-S score ≤4; CGI-SS score ≤2 on part 1 and ≤5 on part 2	Diagnosis other than schizophrenia (DMS-IV-TR); significant medical/neurologic disorder; medically significant abnormal laboratory test or ECG results at screening; patients refractory to AP treatment	Exacerbations of psychotic symptoms/impending relapse: CGI-I score of ≥5 and an increase on any of the 4 PANSS items to a score >4 with an absolute increase of ≥2 or an increase >4 on the combined score of the items; hospitalization; risk of suicide as defined by CGI-SS score of 4 or 5 (part 1) or a score of 6 or 7 (part 2); violent behavior	Aripiprazole once-monthly: 300/400 mg/month Placebo depot	Based on the results of the preplanned interim analysis, the primary endpoint had been achieved, and the study was terminated early. Discontinuation due to other reasons than AEs included subject withdrew consent and other
Fleischhacker (37)	Age: ~41 Male: 60% White: 92% BMI: 27.7 kg/m^2^ Duration (year): 13 Schizoaffective: – Outpatient status: NA *N*=749 53 weeks	DSM-IV diagnosis of schizophrenia for ≥1 year before screening; PANSS total score between 60 and 120 (inclusive); acutely symptomatic at screening; body mass index ≥15.0 kg/m^2^	DSM-IV Axis I diagnosis not schizophrenia; decrease of >25% in PANSS total score (screening to baseline); substance dependence; treatment resistance; neuroleptic malignant syndrome; significant or unstable systemic disease; suicidal/violent behavior; pregnant	Lack of efficacy (not specified)	Paliperidone palmitate: 25/50/75/100 mg/month Risperidone LAI 25/37.5/50 mg/2-week	This study combined acute and maintenance treatment. Discontinuation due to other reasons than AEs included patient choice, loss to follow-up, pregnancy, death, and other
Fleischhacker (39)	Age: ~40–42 Male: 61% White: 58% BMI: 28.8 kg/m^2^ Duration (year): 14 Schizoaffective: – Outpatients only	DSM-IV-TR diagnosis of schizophrenia for ≥3 years; history of symptom exacerbation or relapse when not receiving AP treatment.	Stability criteria: Outpatient status; PANSS total score ≤ 80; Lack of specific psychotic symptoms on the PANSS: score of ≤4 on: conceptual	Impending relapse (not further specified)	Aripiprazole once-monthly: 400 mg/month Aripiprazole oral: 10–30 mg/day Aripiprazole once-monthly	Discontinuation due to other reasons than AEs included patient withdrew consent and other
	Total PANSS score at baseline: not reported *N*=662 38 weeks	Stability criteria: outpatient on part 1 and ≤5 on part 2 status; PANSS total score ≤80; PANSS score of ≤4 on conceptual disorganization, suspiciousness, hallucinatory behavior, and unusual thought content; CGI-S score ≤4; CGI-SS ≤2 on part 1 and ≤5 on part 2	disorganization, suspiciousness, hallucinatory behavior, and unusual thought content; CGI-S score ≤4; CGI-SS ≤2 on part 1 and ≤5 on part 2		50 mg/month (assumed equivalent to placebo)	

a Either study or ‘stable’.

DSM = Diagnostic and Statistical Manual of Mental Disorders; BPRS = Brief Psychiatric Rating Scale (unusual thought content, conceptual disorganization, hallucinations, suspiciousness); CGI-I = Clinical Global Impressions-Improvement of Illness; AP = antipsychotic; ECG = electrocardiogram; CGI-S = Clinical Global Impressions-Severity of Illness; CGI-SS = Clinical Global Impressions-Severity of Suicidality; MTC = mixed treatment comparison, disc's = discontinuations; AE = adverse event.

Few details were provided on the method of randomization of the studies; most of the time only randomization ratios were reported. In all six publications, providers and participants were reported to be blind to treatment. Baseline demographic and disease characteristics were reported in all studies. Similarity between the patient groups was either explicitly stated or could be derived from descriptive statistics. There were no selective reporting issues identified with regard to the primary outcomes in the publications of the included RCTs. Quality assessment of the included studies is presented in the Supplementary file.

### Evidence network

Adopting standard practice, the MTC was conducted at a compound level, that is, extracted data for different therapeutic doses per compound were pooled. Pooling was made after seeing the efficacy results of the corresponding RCTs and concluding that time-to-relapse was similar in these treatment arms.

**Fig. 2:**
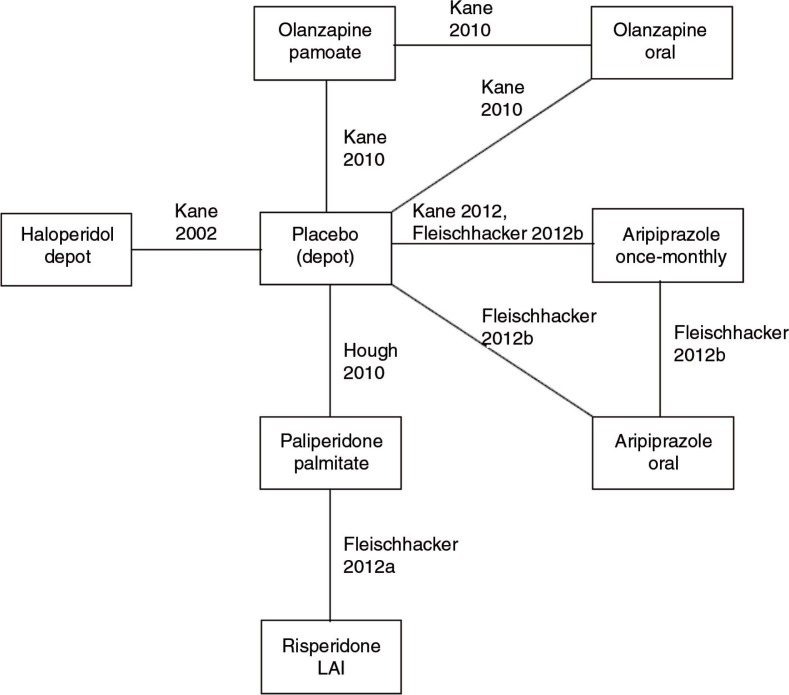
Evidence network. Notes: For the safety analyses, haloperidol depot was not included since no safety data were reported in Ref. (42).

Of the five studies including a placebo arm, only two used ‘true’ placebo in injectable form, whereas three trials used an active antipsychotic depot with subtherapeutic dose (in Fleischhacker et al., 50 mg/month aripiprazole (39, 43); in Kane et al. (41) 45 mg/month olanzapine; in Kane et al. (42) 25 mg/month haloperidol). For the MTC, it was assumed that subtherapeutic dose effect depots were mimicking the efficacy of placebo depots and therefore could be combined. The impact of this assumption was explored in a number of sensitivity analyses. Figure 2 presents the evidence network, whereas Table 2 displays the data that were extracted from the RCTs and that were used for the MTC analyses.

**Table 2:** Reported efficacy, tolerability, and safety data used in the MTC

Study name	Treatment arms	Time horizon (weeks)	Relapses	AE-related disc's	Non-AE-related disc's	Completers	Total Nr of patients	Nr of patients experiencing >7% weight gain (total Nr of patients)	Nr of patients experiencing EPS (total Nr of patients)
Kane (42)	Haloperidol depot^a^	52	17	1	18	44	80	NA	NA
	Placebo^b^	52	15	0	2	8	25	NA	NA
Kane (41)	Olanzapine pamoate^c^	24	68	21	91	419	599	100 (599)	52 (599)
	Olanzapine oral	24	23	8	33	258	322	68 (322)	28 (322)
	Placebo^b^	24	42	6	20	76	144	12 (144)	12 (144)
Hough 2010 (38)	Paliperidone palmitate	31	36	3	28	139	206	12 (206)	21 (206)
	Placebo	42	97	2	27	78	204	6 (204)	12 (204)
Kane (40)	Aripiprazole once-monthly	52	27	9	31	202	269	17 (269)	45 (269)
	Placebo	52	53	5	15	61	134	7 (134)	14 (134)
Fleischhacker (37)	Paliperidone palmitate	53	95	29	100	155	379	50 (346)	67 (379)
	Risperidone LAI	53	56	25	105	184	370	52 (338)	76 (370)
Fleischhacker (39)	Aripiprazole once-monthly	38	22	8	39	196	265	42 (265)	52 (265)
	Aripiprazole oral	38	21	7	60	178	266	43 (266)	46 (266)
	Placebo^b^	38	29	7	34	61	131	8 (131)	18 (131)

disc's = discontinuations; EPS = extrapyramidal symptoms; MTC = mixed treatment comparison; Nr = number.

a Pooled dose approach (200, 100, and 50 mg once-monthly, respectively).

b Subtherapeutic dose of the active long-acting injectable comparator.

c Pooled dose approach (150 and 300 mg biweekly, and 405 mg four-weekly).

### Data synthesis

#### Efficacy and tolerability analysis

The data showed a considerably better fit for the random effects model (DIC=365.1) than for the fixed effects model (DIC=389.1), therefore the random effects model was chosen. In the following results of the random effects model are described. All diagnostic plots showed convergence.

Table 3 presents the estimated HRs for each outcome (relapse, discontinuation due to AEs, and discontinuation due to other reasons) for each active treatment relative to placebo. HRs for aripiprazole OM versus its LAI comparators are presented in the Supplementary file. Compared to placebo, the rate of relapse was numerically smallest for aripiprazole OM (mean HR=0.26, 95% credibility interval [CI] 0.12–0.51) and risperidone LAI (HR=0.27). The HR of relapse for other drugs was 0.44 (paliperidone palmitate), 0.39 (olanzapine pamoate), 0.37 (haloperidol depot). 95% CrIs indicated that there was substantial uncertainty around the estimates, and therefore no significant differences between the HRs were observed.

**Table 3:** Hazard ratios and 26-week probabilities for efficacy outcomes, random effects mixed treatment comparison

Treatment	HR	LCrI	UCrI	26-week probability (%)	LCrI (%)	UCrI (%)	Treatment is best (%)
	Relapse						
Placebo	1.00			27.6	18.5	39.3	0.0
Aripiprazole once-monthly	0.26	0.12	0.51	8.4	3.3	17.1	9.4
Haloperidol depot	0.37	0.10	0.97	9.6	1.9	25.4	16.7
Olanzapine pamoate	0.40	0.13	0.91	11.5	3.5	27.3	2.6
Paliperidone palmitate	0.44	0.14	1.00	12.2	3.4	28.7	0.9
Risperidone LAI	0.27	0.05	0.84	7.7	1.2	23.8	34.5
Aripiprazole oral	0.34	0.10	0.84	10.3	2.9	25.3	9.1
Olanzapine oral	0.23	0.07	0.55	7.3	2.0	18.3	26.9
	Discontinuation due to AEs						
Placebo	1.00			2.5	1.0	5.0	0.3
Aripiprazole once-monthly	0.74	0.17	2.12	2.1	0.3	6.8	9.2
Haloperidol depot	4.26	0.04	25.56	5.5	0.1	43.9	29.0
Olanzapine pamoate	1.24	0.14	4.03	3.0	0.3	11.8	5.9
Paliperidone palmitate	4.69	0.16	22.24	8.5	0.3	46.9	2.3
Risperidone LAI	5.92	0.09	26.91	8.4	0.2	53.0	9.0
Aripiprazole oral	0.68	0.07	2.33	1.9	0.1	7.4	25.3
Olanzapine oral	0.77	0.09	2.92	2.2	0.2	9.2	18.9
	Discontinuation due to other than AEs						
Placebo	1.00			11.3	5.8	18.8	0.6
Aripiprazole once-monthly	0.80	0.25	1.55	8.8	2.6	21.1	27.1
Haloperidol depot	3.29	0.39	13.96	27.8	3.9	83.5	3.9
Olanzapine pamoate	1.27	0.27	3.56	14.0	2.9	39.3	5.4
Paliperidone palmitate	1.49	0.28	4.14	14.9	2.9	42.5	4.9
Risperidone LAI	2.00	0.18	6.59	16.8	1.7	58.1	12.1
Aripiprazole oral	0.95	0.21	2.66	11.6	2.4	33.6	16.0
Olanzapine oral	0.85	0.17	2.27	9.7	1.9	29.3	30.0
	Continuing treatment						
Placebo				58.7	46.6	68.4	0.0
Aripiprazole once-monthly	N/A	N/A	N/A	80.8	65.6	89.6	28.8
Haloperidol depot	N/A	N/A	N/A	57.1	5.3	85.3	2.9
Olanzapine pamoate	N/A	N/A	N/A	71.5	42.9	86.7	1.9
Paliperidone palmitate	N/A	N/A	N/A	64.4	24.4	83.8	0.6
Risperidone LAI	N/A	N/A	N/A	67.1	14.3	89.3	9.3
Aripiprazole oral	N/A	N/A	N/A	76.3	50.3	89.6	15.1
Olanzapine oral	N/A	N/A	N/A	80.8	57.4	91.8	41.5

HR=hazard ratio; LCrI=95% lower credible interval; UCrI=95% upper credible interval.

The HR of discontinuations due to AEs was estimated to be lower than 1 for aripiprazole OM (HR=0.74, 95% CI 0.17–2.12) and higher than 1 for all other LAIs (paliperidone palmitate: HR = 4.69, risperidone LAI: HR = 5.92, olanzapine pamoate: HR = 1.24, haloperidol depot: HR = 4.26), although all CrIs included 1 and were overlapping, so differences were not significant. Similarly, the HR of discontinuation due to reasons other than AEs was estimated to be lower than 1 for aripiprazole OM (HR = 0.80, 95% CrI 0.25–1.55) and higher than 1 for the other LAIs (paliperidone palmitate: HR=1.49, risperidone LAI: HR=2.00, olanzapine pamoate: HR=1.27, haloperidol depot: HR = 3.29), again with all CrIs including 1 and overlapping.

Table 3 presents the estimated probabilities for each outcome and each treatment including placebo depot. Additionally, the probability that a treatment is the best therapeutic option for a particular outcome type is also shown. Among depots, risperidone LAI, aripiprazole OM, haloperidol depot, olanzapine pamoate, and paliperidone palmitate had an estimated relapse risk of 7.7, 8.4, 9.6, 11.5, and 12.2%, respectively. The relapse risk for placebo depot was estimated at 27.6%. The width of the 95% CrIs indicated that there is substantial uncertainty around these estimates, with no significant differences between treatments. This uncertainty was also reflected in the small ‘best maintenance treatment option’ probabilities, with none significantly outperforming the others; the probability of being the best in ranking with respect to the risk of relapse for risperidone LAI, haloperidol depot, aripiprazole OM, olanzapine pamoate, and paliperidone palmitate was 34.5, 16.7, 9.4, 2.6, and 0.9%, respectively. Placebo depot had 0% probability of being the best relapse prevention treatment.

The risk of discontinuing treatment due to AEs was 8.5% for patients receiving paliperidone palmitate, 8.4% for those on risperidone LAI, and 5.5% for patients on haloperidol depot. For patients receiving aripiprazole OM or olanzapine pamoate, the risk of discontinuation treatment due to AEs was estimated at 2.1 and 3.0%, respectively, close to the rates predicted for placebo (2.5%), although no significant differences emerged. Similarly, although CrIs were large around the estimates, it was predicted that 8.8% of aripiprazole OM patients would discontinue aripiprazole OM treatment due to reasons other than AEs while corresponding figures were 27.8% for haloperidol depot, 16.8% for risperidone LAI, 14.9% for paliperidone palmitate, and 14.0% for olanzapine pamoate.

Due to low relapse risk and discontinuation probabilities, aripiprazole OM was estimated to have a probability of continuing maintenance treatment over a 26-week period of 80.8%, with olanzapine pamoate having a treatment continuation probability of 71.5%, risperidone LAI 67.1%, paliperidone palmitate 64.4%, and haloperidol depot 57.1%. No statistically significant difference between the treatments was found.

**Fig. 3:**
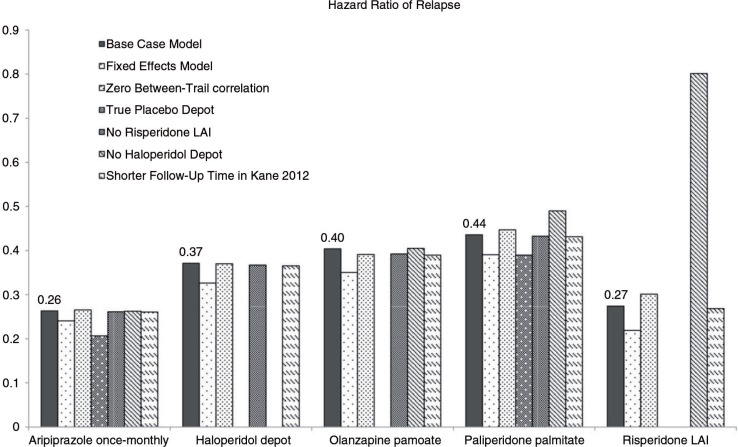
Sensitivity analyses results: Hazard ratio of relapse in alternative models.

To examine the robustness of the model results, various sensitivity analyses were performed. Results are presented in terms of HR of relapse in Fig. 3. Overall, the difference in HRs was less than 0.1 compared to those of the base case analyses. The results of the sensitivity analyses indicated that the impact of these changes in assumption was minimal on the outcomes, highlighting the robustness of the estimated risks of relapse in the base case analysis. Particularly, the results of the analysis limited to only those studies that used true placebo injections (i.e., no subtherapeutic dose antipsychotics) were considered as an indication that the efficacy of treatments with subtherapeutic dose can be assumed to be similar to that of placebo depot.

#### Safety analyses

For both safety outcomes, the data showed slightly better fit of the fixed effects model (DIC = 82.5 for clinically relevant weight gain and 83.3 for EPS) than of the random effects models (DIC = 82.7 for clinically relevant weight gain and 84.9 for EPS); therefore, the fixed effects models were chosen. In case of both analyses, diagnostic tests indicated model convergence.

Table 4 presents the estimated odds ratios and 26-week probability of experiencing clinically relevant weight gain and EPS, separately, for each antipsychotic. Compared to placebo, active treatments increased the risk of these AEs. In terms of clinically relevant weight gain, aripiprazole OM, paliperidone palmitate, olanzapine pamoate, and risperidone LAI was associated with a risk of 11.2% (95% CrI: 6.5–18.4%), 11.9% (95% CrI: 4.2–26.1%), 12.0% (95% CrI: 6.6–20.3%), and 12.8% (95% CrI: 4.2–29.1%), respectively. In terms of EPS, olanzapine pamoate, aripiprazole OM, paliperidone palmitate, and risperidone LAI was associated with a risk of 10.1% (95% CrI: 5.5–17.6%), 14.3% (95% CrI: 9.7–20.4%), 16.3% (95% CrI: 8.2–28.4%), and 19.1% (95% CrI: 9.0–34.5%), respectively. The 95% CrIs were large indicating substantial uncertainty around the point estimates.

**Table 4:** Odds ratios and 26-week probabilities for safety outcomes, fixed effects mixed treatment comparison

Treatment	OR	LCrI	UCrI	26-week probability (%)	LCrI (%)	UCrI (%)	Treatment is best (%)
	Clinically significant (>7%) weight gain						
Placebo	1			5.4	3.6	7.2	89.7
Aripiprazole once-monthly	2.24	1.22	3.96	11.2	6.5	18.4	0.3
Haloperidol depot	–			–	–	–	–
Olanzapine pamoate	2.42	1.25	4.48	12.0	6.6	20.3	0.3
Paliperidone palmitate	2.47	0.78	6.21	11.9	4.2	26.1	4.5
Risperidone LAI	2.70	0.77	7.22	12.8	4.2	29.1	4.9
Aripiprazole oral	2.45	1.25	4.48	12.1	6.6	20.3	0.4
Olanzapine oral	3.24	1.64	6.14	15.3	8.5	25.9	0
	EPS						
Placebo	1			9.1	6.8	11.5	37.9
Aripiprazole once-monthly	1.68	1.07	2.55	14.3	9.7	20.4	0.3
Haloperidol depot	–			–	–	–	–
Olanzapine pamoate	1.14	0.58	2.12	10.1	5.5	17.6	24.0
Paliperidone palmitate	1.99	0.89	3.94	16.3	8.2	28.4	2.5
Risperidone LAI	2.43	0.99	5.23	19.1	9.0	34.5	0.8
Aripiprazole oral	1.43	0.83	2.33	12.5	7.7	19.0	6.4
Olanzapine oral	1.14	0.54	2.24	10.2	5.1	18.4	28.1

OR=odds ratio; LCrI=95% lower credible interval; UCrI=95% upper credible interval.

## Discussion

The present study provided an overview of the relevant literature of selected LAI antipsychotic medications used in maintenance treatment of schizophrenia and compared aripiprazole OM with the other LAIs. Only RCTs were considered, which are the gold standard of clinical evidence minimizing the risk of confounding factors and allowing the comparison of the relative efficacy of interventions. During the literature review efficacy, tolerability, and safety data were searched. A random effect competing risk MTC model was built on efficacy and tolerability endpoints, whereas standard fixed effects binary models were used for the safety outcomes. To our knowledge, this is the first MTC assessing comparative efficacy, safety, and tolerability outcomes of LAI antipsychotics in the maintenance treatment of schizophrenia.

For the efficacy and tolerability analysis, six relevant RCTs were identified as eligible for inclusion. These studies formed an evidence network including five LAI antipsychotics (aripiprazole, olanzapine, risperidone, paliperidone, and haloperidol), placebo injections, and complemented with two oral antipsychotics (aripiprazole and olanzapine) as active reference. While haloperidol is a first-generation antipsychotic, it remains an important treatment in developing countries. There were comparable efficacy and tolerability profiles; relapse probability, treatment discontinuation probabilities both due to AEs and due to other reasons; and treatment continuation probability over 6 months across the long-acting preparations. The point estimates from the MTCs suggested that aripiprazole OM is similarly efficacious and tolerable in the management of relapse prevention as other LAIs with a numerical but non-statistically significant advantage over other LAIs in terms of discontinuation due to AEs and due to other reasons than AEs. Likewise, the safety analyses indicated similar AE risks profiles of treatments with non-statistically significant differences between the LAIs.

### Previous studies

A number of meta-analyses have been conducted previously that assessed LAI antipsychotics. Kishimoto et al. (23) compared LAIs to oral antipsychotics in terms of study-defined relapse, all-cause discontinuation, discontinuation due to AEs, hospitalization, and non-adherence, separately, based on 21 RCTs. Results indicated that LAIs were similar to orals for relapse prevention (relative risk [RR] = 0.93, 95% confidence interval [CI]: 0.80–1.08). Similarly, Leucht et al. (26) compared depot with oral antipsychotics in schizophrenia in terms of relapse, rehospitalization, non-adherence and dropout due to any reason, inefficacy of treatment, and AEs based on 10 RCTs of ≥12 months of duration. Contrary to the findings of Kishimoto et al., Leucht et al. suggested that depot formulations significantly reduced the relapses (RR=0.70, 95% CI 0.57–0.87). Due to a number of methodological problems, however, the evidence was admittedly subject to possible bias. Fusar-Poli et al. (24) tested the efficacy and safety of second-generation long-acting antipsychotic injections (SGLAI) versus placebo and oral antipsychotics based on 13 RCTs. SGLAIs were more effective than placebo injections in reducing PANSS scores (Hedge's *g*=0.34, 95% CI 0.25–0.43), but no statistically significant difference was observed if SGLAIs were compared to oral antipsychotics (Hedge's *g*=0.07, 95% CI −0.07–0.21). Kishimoto et al. (44) conducted a meta-analysis of mirror-image studies of all LAIs that compared the period of oral antipsychotic treatment with subsequent period of LAI treatment. Due to the inherent study design, mirror-image studies have been suggested to better reflect the real-world impact of LAIs than RCTs, which might enroll disproportionate number of patients with better treatment adherence. In this meta-analysis, LAIs showed superiority over oral antipsychotics in preventing hospitalization (RR = 0.43, 95% CI 0.35–0.53, based on 16 studies). Finally, Lafeuille et al. (45) evaluated the impact of LAIs versus oral antipsychotics on hospitalizations using results of 58 studies. When assessing the percentage decrease in hospitalization rates before and after treatment initiation for matched time periods, LAIs were found to be associated with larger reductions than oral antipsychotics (P value 0.027). When assessing the absolute rate of hospitalization during follow-up, no significant difference between LAIs and oral antipsychotics was observed (P value 0.077). All these above studies commonly made the assumption that the efficacy of particular LAI antipsychotics is similar, and that they can be merged into a single class of treatment. Despite the differences between previous meta-analyses and the present study, our MTC results may confirm such an assumption since no statistically significant differences could be shown between the LAIs.

### Validity of the MTC

In general, the validity of an MTC depends to a large extent on the characteristics of the individual studies that it is based on. Particularly, the included studies must be sufficiently homogenous in terms of trial design and patient population, in order to be comparable and to prevent bias potentially arising from differences across trials with respect to treatment effect modifiers. To this end, rigorous inclusion and exclusion criteria were defined to identify eligible studies during the literature review. Only studies that had sufficiently long follow-up time, that randomized patients at the start of the maintenance phase, and that recruited similar patient populations were eligible for inclusion. If there was no definition of ‘stable patient’ provided in the trial documentation, other measures like PANSS scores and outpatient status were checked. While such study selection procedure resulted in the exclusion of most studies, and as a consequence the included studies reflected only a small subset of the real-world patient population, it ensured that all eligible RCTs had a similar and comparable patient population. Failure to exclude ineligible studies would have introduced potential bias in the analyses because treatment effects may differ between these patient populations, and thus relative treatment effects of heterogeneous populations would have been compared.

The average baseline PANSS score in Fleischhacker (37) (approximately 81–82) was at the lower end of the range of average baseline PANSS scores typically measured in patients with acute schizophrenia (46–48). Thus, it may be conjectured that the difference in patient characteristics in terms of PANSS scores between stable patients and the study population of Fleischhacker (37) was not that large. Furthermore, although in Fleischhacker (37) both paliperidone palmitate and risperidone LAI improved schizophrenia symptom severity and reduced severity of illness, paliperidone palmitate did not demonstrate comparable efficacy to risperidone LAI. This has been attributed to the initiation dosing strategy employed, which used a low paliperidone palmitate dose (37). Consequently, the inclusion of this study into the evidence network, that is, lower relative efficacy of paliperidone palmitate against risperidone LAI than in real clinical practice, tended to underestimate the relative efficacy and safety between aripiprazole OM and risperidone LAI. On the other hand, the inclusion of Fleischhacker (37) did not affect the comparison between aripiprazole OM and paliperidone palmitate because it added information on the relative efficacy to the evidence base between risperidone LAI and paliperidone palmitate only.

Some residual heterogeneity could have remained between the study populations. Due to unavailable data, the study selection criteria did not consider heterogeneity of patients in terms of regional differences. Such consideration may be important because recent exploratory analyses have suggested that genetic variation due to different ancestry may have a predictive effect on how well patients respond to antipsychotic medication (49). Unfortunately, most RCTs from which input data for the MTC were extracted did not allow subgroup analyses by country or region. Nevertheless, except for Hough et al. for which the study sites were not revealed, the RCTs recruited patients mostly from Western populations (38). Thus, any potential differences in the regional representation of patients across RCTs were likely not to be significant and could hardly have any effect on the results of the MTC.

The definition of relapse used across studies could potentially bias the MTC results. Currently, there are no established criteria that define relapse and international or national guidelines do not provide a definition either. The RCTs identified during the literature review eligible for the MTC used a slightly different relapse measure, such as symptomatic exacerbation, psychotic exacerbation, impending relapse, or hospitalization (see details in Table 1). Nevertheless, it is not expected that differences in the definition of relapse were substantial since most RCTs covered a relatively short period of time, that is, 2010–2012 with the exception of Kane et al. published in 2002. It was suspected that the identification of relapse in clinical practice is likely not to change over such a short time frame and that the definition of relapse in the RCTs reflected that. Furthermore, previously published meta-analyses, in which the patient populations were less homogeneous, also made similar assumptions on relapse (24, 50).

For the systematic literature review, trials published before May 2013 were searched for, and hence the MTCs were based on the available evidence for this period. To determine whether relevant new evidence (i.e., RCTs assessing LAI antipsychotic in the maintenance treatment of schizophrenia in stable patients) has been published since May 2013, a targeted literature review in PubMed was performed up to May 2015. No randomized clinical trial was identified that met the inclusion and exclusion criteria and that could have been added to the evidence network.

## Conclusions

The systematic literature review identified six studies from which data could be derived for the MTCs. The analyses suggested that aripiprazole OM, risperidone LAI, paliperidone palmitate, olanzapine pamoate, and haloperidol are similarly efficacious. Risperidone LAI was associated with a numerical advantage in relapse rate, while aripiprazole OM had a numerical advantage in terms of discontinuation due to AEs and due to reasons other than AEs. However, no statistically significant differences were shown in any of these measures. A similar safety profile of the considered LAIs was demonstrated. Future research should focus on conducting real-world studies to assess the validity of these results in clinical practice.

## Supplementary Material

Efficacy, tolerability, and safety of aripiprazole once-monthly versus other long-acting injectable antipsychotic therapies in the maintenance treatment of schizophrenia: a mixed treatment comparison of double-blind randomized clinical trials
