# Supplementary material for: Efficacy, tolerability, and safety of aripiprazole once-monthly versus other long-acting injectable antipsychotic therapies in the maintenance treatment of schizophrenia: a mixed treatment comparison of double-blind randomized clinical trials
Source: J Mark Access Health Policy. 2015 Sep 10;3:10.3402/jmahp.v3.27208. doi: 10.3402/jmahp.v3.27208 (PMC4802697; doi:10.3402/jmahp.v3.27208)
Supplement: Efficacy, tolerability, and safety of aripiprazole once-monthly versus other long-acting injectable antipsychotic therapies in the maintenance treatment of schizophrenia: a mixed treatment comparison of double-blind randomized clinical trials [file JMAHP-3-27208-s001.pdf]

SUPPLEMENTARY MATERIAL

1. Study inclusion criteria for the systematic literature review extension to May 2013

Criteria	Description	Notes
Population	• Age: adults (≥ 18 years)<br>• Gender: any<br>• Race: any<br>• Stable disease	Schizophrenia can occur both in men and women with equal prevalence. Race or ethnicity does not influence the prevalence of schizophrenia. Therefore, adults of any race or gender diagnosed with schizophrenia were included in this review. Stable disease may be measured differently but a PANSS score of around 60 and outpatient status generally indicate stable disease.
Disease	Diagnosis of schizophrenia that was not treatment resistant	Schizoaffective disorder was not considered in this review.
Intervention	• Aripiprazole once-monthly<br>• Olanzapine pamoate<br>• Paliperidone palmitate<br>• Risperidone LAI<br>• Haloperidol depot	
Comparator	• Any other included intervention<br>• Placebo	These comparators were selected to enable both direct and indirect comparisons between the interventions of interest.
Study design	Double-blind / triple-blind randomized control trials	Randomized controlled trials are the gold standard of clinical evidence, minimizing the risk of confounding and allowing the comparison of the relative efficacy of interventions. Therefore only these studies were included.
Treatment phase	Maintenance treatment	Inclusion was restricted to maintenance treatment in order to align with the expected marketing authorization for aripiprazole once-monthly
Language restrictions	English only	The restriction would not limit results substantially as most of the research is published in English language journals.
Sample size	≥10 participants	The sample size of the included studies was in line with the NICE clinical review protocol (27)
Study duration	Long-term follow-up (at least 6 months)	The study duration of the included studies was in line with the NICE clinical review protocol (27).

2. Study exclusion criteria for the systematic literature review extension to May 2013

Criteria	Description	Notes
Subgroup analysis	• No subgroup analysis for disease of interest<br>• No subgroup analysis for adult population	Studies with no subgroup data for the disease, adult population, or maintenance treatment in stable patients were not included, since these studies would introduce heterogeneity into the review.
Outcome of interest	Studies not reporting an outcome interest	Studies which do not report outcomes of interest would not feature in any analyses and were therefore excluded from extraction

3. PRISMA Flow diagram

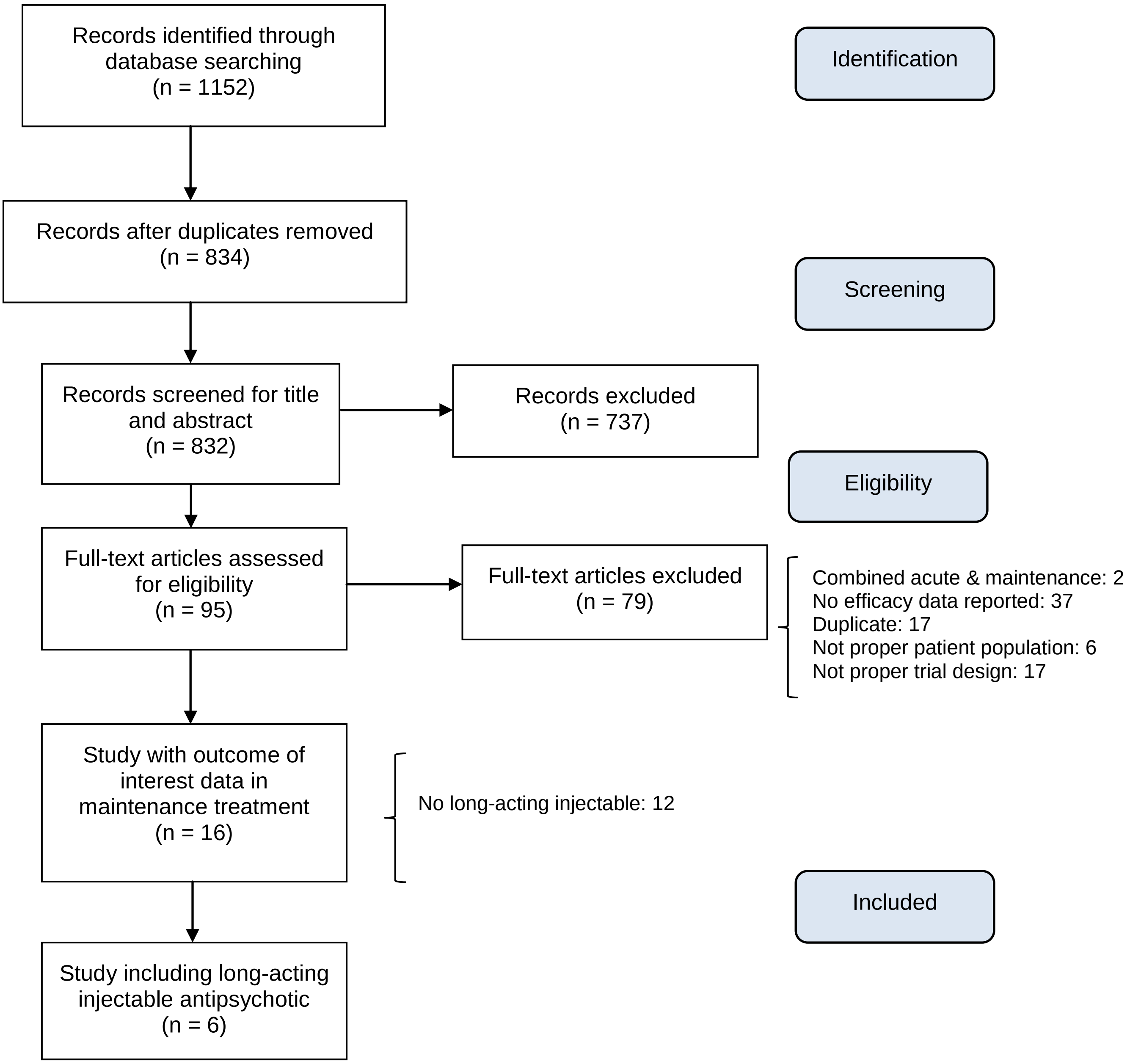

Note: because the literature search strategy of this study adopted the one used for the NICE schizophrenia clinical guidance, oral formulations and depot formulations were both considered throughout the search. Studies of oral formulation antipsychotics were excluded after full text review.

4. Quality assessment of the included RCTs

Trial	Fleischhacker 2012a	Fleischhacker 2002b	Hough 2010	Kane 2002	Kane 2010	Kane 2012
Was randomisation carried out appropriately?	Yes (“computer based randomization scheme”)	Unclear	Yes (“via a sponsor-prepared computer-generated randomization scheme; assigned by an interactive voice response system”)	Unclear	Unclear	Unclear
Was the concealment of treatment allocation adequate?	Unclear	Unclear	Unclear	Unclear	Unclear	Unclear
Were the groups similar at the outset of the study in terms of prognostic factors?	Yes (“The demographic and baseline characteristics were similar between treatment groups”)	Yes (“Baseline demographic characteristics were similar between randomized treatment groups”)	Yes (Age and sex at baseline were similar. Furthermore “The mean PANSS total scores and CGI-S scores at transition phase baseline suggested that the population was symptomatically stable and at double-blind baseline indicated adequate symptom control during the maintenance phase”)	Yes (Patients were similar in terms of age, sex, duration of illness, BPRS total score)	Yes (“No statistically significant differences were observed for baseline physical characteristics and illness history”)	Yes (“Baseline demographic and disease characteristics of randomized subjects were similar between treatment groups”)

5. Supplementary results

Table 5. Efficacy and tolerability outcomes

	Mean	Median	2.50%	97.50%
**Aripiprazole once-monthly versus Olanzapine pamoate**				
Relapse	76.18	0.70	0.214	2.43
Disc. AE	2.09	0.75	0.099	6.176
Disc. Other	2.77	0.61	0.136	2.953
**Aripiprazole once-monthly versus Paliperidone palmitate**				
Relapse	0.84	0.63	0.186	2.142
Disc. AE	1.00	0.34	0.020	4.951
Disc. Other	14.93	0.55	0.111	2.852
**Aripiprazole once-monthly versus Risperidone LAI**				
Relapse	1713.00	1.15	0.234	5.446
Disc. AE	6.77	0.42	0.018	9.042
Disc. Other	2.18E+09	0.56	0.078	4.51
**Aripiprazole once-monthly versus Haloperidol depot**				
Relapse	1.061	0.791	0.207	3.074
Disc. AE	4.314	0.864	0.018	22.19
Disc. Other	16.35	0.302	0.035	2.005

Table 6. Safety outcomes

	Mean	Median	2.50%	97.50%
**Aripiprazole once-monthly versus Olanzapine pamoate**				
Weight gain	1.029	0.938	0.383	2.215
EPS	1.65	1.53	0.68	3.32
**Aripiprazole once-monthly versus Paliperidone palmitate**				
Weight gain	1.203	1.004	0.297	3.251
EPS	0.97	0.90	0.37	2.07
**Aripiprazole once-monthly versus Risperidone LAI**				
Weight gain	1.149	0.941	0.259	3.227
EPS	0.82	0.74	0.28	1.86

Forest plots of efficacy and tolerability outcomes

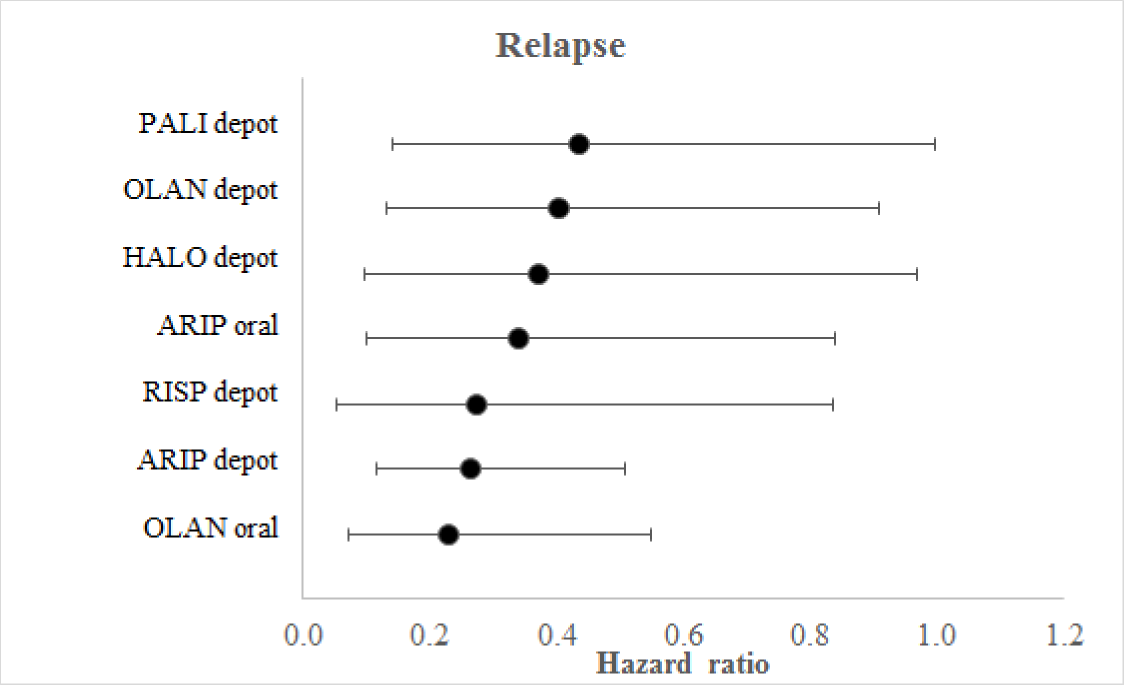

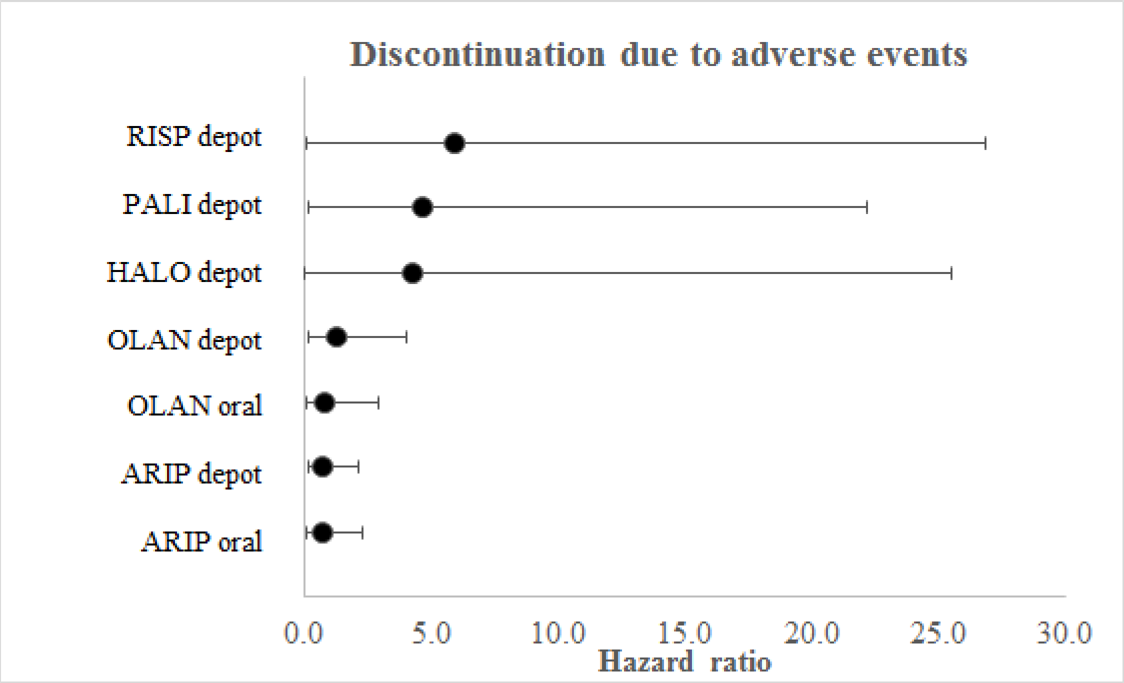

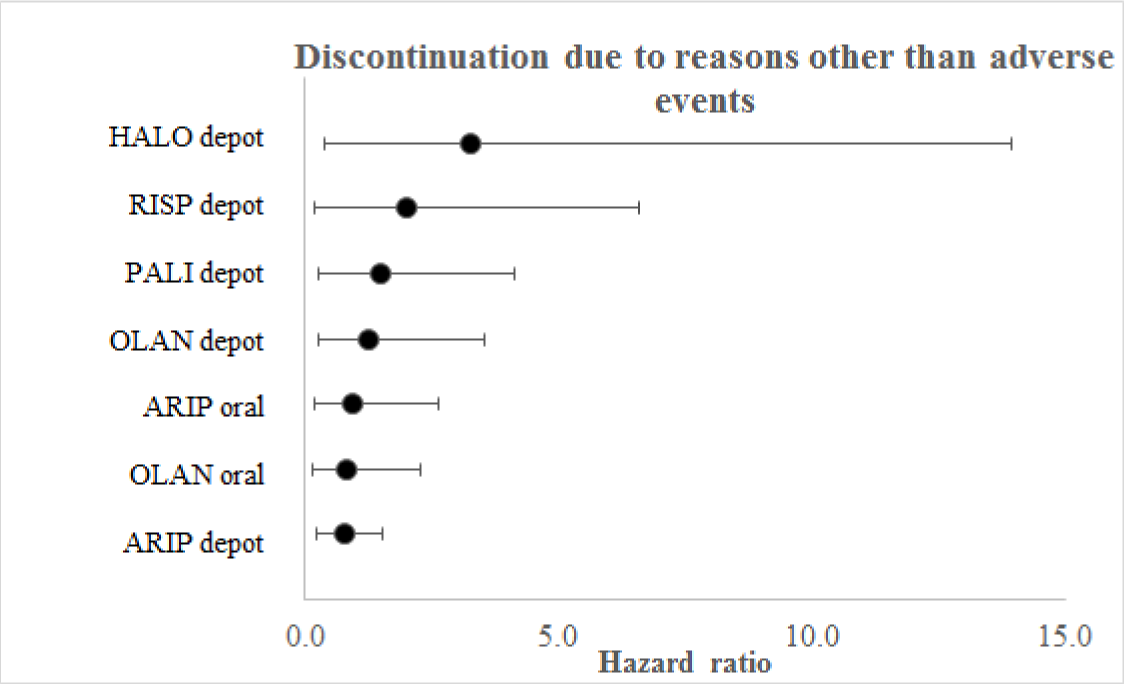

6. WinBugs code for the efficacy and tolerability analysis

Model

```
model{

# Ni = number of arms
# Nk = number of treatments
# Nj = number of trials
# Nib = number of placebo arms

# Code for treatment effects relative to placebo (treatment 1)
for(i in 1:Ni) {
       # multinomial likelihood
       r[i,1:4] ~ dmulti(p[i,1:4],n[i])
       # sum of the hazard rates for the 3 discontinuation outcomes
       slam[i] <- sum(lam[,i])
       for (m in 1:3) {
              # probability of reaching each discontinuation outcome
              p[i,m] <- lam[m,i] * (1-exp(-slam[i]*w[i]/26)) / slam[i]
              # log hazard rates for each arm, each outcome
              log(lam[m,i]) <- theta[m,i]
              theta[m,i] <- mu[m,s[i]] + delta[m,i]*(1-equals(t[i],b[i]))
              # random effects model for log hazard ratios with correction for three-
arm trials
              delta[m,i] ~ dnorm(md[m,i],taud[m,i])
              taud[m,i] <- tau[m] * (1 + equals(arm[i],3) /3)
              md[m,i] <- d[m,t[i]] - d[m,b[i]] + equals(arm[i],3) * sw[m,i]
       }
       # probability of continuing treatment
       p[i,4] <- 1- sum(p[i,1:3])
}

# Correction factor for 3-arm trials
```

```
for (m in 1:3) {
	sw[m,1] <- 0
	for (i in 2:Ni) { sw[m,i] <- (delta[m,i-1] - d[m,t[i-1]] + d[m,b[i-1]] ) /2 }
}

# Code for absolute effects of placebo (treatment 1)
for (i in 1:Nib) {
	rb[i,1:4] ~ dmulti(pb[i,1:4],nb[i])
	for (m in 1:3) {
		pb[i,m] <- lamb[m,i] * (1-exp(-slamb[i]*wb[i]/26)) / slamb[i]
		log(lamb[m,i]) <- mub[m,sb[i]]
	}
	slamb[i] <- sum(lamb[,i])
	pb[i,4] <- 1- sum(pb[i,1:3])
}

# Priors
for (m in 1:3) {
	d[m,1] <- 0
	for (k in 2:Nk) {
		# priors for treatment effects
		d[m,k] ~ dnorm(0,.0001)
		log(hazr[m,k]) <- d[m,k] # hazard ratios
	}
	# priors for baselines
	for (j in 1:Nj) { mu[m,j] ~ dnorm(0,.0001) }
}
for (m in 1:3) {
	# variance of the log hazard ratios
	tau[m] <- pow(sdb[m],-2) * pow(2*(1-rho[m]),-.5)
}
for (m in 1:3) {
	# priors for treatment effects and baselines for placebo
	for (j in 1:Nib) { mub[m,j] ~ dnorm(mb[m],prb[m]) }
	mb[m] ~ dnorm(0,.0001)
}
for (m in 1:3) {
	# priors for between-trial variation
	prb[m] ~ dgamma(.1,.1)
	sdb[m] <- pow(prb[m],-.5)
	rho[m] ~ dunif(0,1)
}

# Code for estimated absolute probabilities at 26 weeks
for (m in 1:3) {
	for (k in 1:Nk) {
		theta26[m,k] <- mb[m] + d[m,k]
		log(lam26[m,k]) <- theta26[m,k]
		p26[m,k] <- lam26[m,k] * (1-exp(-slam26[k])) / slam26[k]
	}
}
for (k in 1:Nk) {
	# probability of discontinuing treatment
	slam26[k] <- sum(lam26[1:3,k])
	# probability of continuing treatment
	p26[4,k] <- 1-sum(p26[1:3,k])
}

# Code for estimating that each treatment is the best option
for (k in 1:Nk){
	for (m in 1:4){
		# rank of each treatment from smallest to largest
		rank26[m,k] <- rank(p26[m,],k)
```

```
       }
       # record whether best (i.e. smallest probability, rank 1) for each
discontinuation outcome
       for (m in 1:3) { best[m,k] <- equals(rank26[m,k],1) }
       # record whether best (i.e. largest probability, rank Nk) for continuation
outcome
       best[4,k] <- equals(rank26[4,k],Nk)
}
# probabilities of being ranked jth (j=1,...,Nk) for each outcome
for (k in 1:Nk){
       # record whether jth best w.r.t. relapse
       for (j in 1:Nk) { rankREL[j,k] <- equals(rank26[1,k],j) }
       # record whether jth best w.r.t. discontinuation due to adverse events
       for (j in 1:Nk) { rankAE[j,k] <- equals(rank26[2,k],j) }
       # record whether jth best w.r.t. discontinuation due to other reasons
       for (j in 1:Nk) { rankOTHER[j,k] <- equals(rank26[3,k],j) }
       # record whether jth worst w.r.t. continuation (NOTE: REVERSED ORDER)
       for (j in 1:Nk) { rankCONT[j,k] <- equals(rank26[4,k],j) }
}

}
```

Data

```
list(

Nk = 8, # treatments
Nj = 6, # trials
Ni = 14, # arms

r = structure(.Data =
c(22,8,39,196,21,7,60,178,29,7,34,61,36,3,28,139,97,2,27,78,18,2,19,45,16,1,3,9,42,6,2
0,76,68,21,91,419,23,8,33,258,27,9,31,202,53,5,15,61,56,25,105,184,95,29,100,155),
.Dim = c(14,4)),

n=c(265,266,131,206,204,84,29,144,599,322,269,134,370,379),
b =c(1,1,1,1,1,1,1,1,1,1,1,1,6,6),
t = c(2,7,1,5,1,3,1,1,4,8,2,1,6,5),
s = c(1,1,1,2,2,3,3,4,4,4,5,5,6,6),
w  = c(38,38,38,31,42,52,52,24,24,24,52,52,53,53),
arm = c(1,2,3,1,2,1,2,1,2,3,1,2,1,2),

# For baseline: placebo (treatment 1)

Nib = 5, # placebo arms
rb = structure(.Data = c(29,7,34,61,97,2,27,78,16,1,3,9,42,6,20,76,53,5,15,61), .Dim =
c(5,4) ),
nb = c(131,204,29,144,134),
wb  = c(38,42,52,24,52),
sb = c(1,2,3,4,5)
)
```

Initial values #1

```
# initial values 1
list(
delta=structure(.Data=c(0,0,0,0,0, 0,0,0,0,0, 0,0,0,0,
       0,0,0,0,0, 0,0,0,0,0, 0,0,0,0,
       0,0,0,0,0, 0,0,0,0,0, 0,0,0,0),.Dim=c(3,14)),
d=structure(.Data=c(NA,0,0,0,0, 0,0,0,
       NA,0,0,0,0, 0,0,0,
       NA,0,0,0,0, 0,0,0),.Dim=c(3,8)),
```

```
mu=structure(.Data=c(0,0,0,0,0, 0,
       0,0,0,0,0, 0,
       0,0,0,0,0, 0),.Dim=c(3,6)),
mub=structure(.Data=c(0,0,0,0,0,
       0,0,0,0,0,
       0,0,0,0,0),.Dim=c(3,5)),
mb=c(0,0,0),
prb=c(1,1,1),
rho=c(.2,.2,.6)
)
```

Initial values #2

```
# initial values 1
list(
delta=structure(.Data=c(0,0,0,0,0, 0,0,0,0,0, 0,0,0,0,
       0,0,0,0,0, 0,0,0,0,0, 0,0,0,0,
       0,0,0,0,0, 0,0,0,0,0, 0,0,0,0),.Dim=c(3,14)),
d=structure(.Data=c(NA,-1,-1,-1,-1, -1,-1,-1,
       NA,-1,-1,-1,-1, -1,-1,-1,
       NA,-1,-1,-1,-1, -1,-1,-1),.Dim=c(3,8)),
mu=structure(.Data=c(-1,-1,-1,-1,-1, -1,
       -1,-1,-1,-1,-1, -1,
       -1,-1,-1,-1,-1, -1),.Dim=c(3,6)),
mub=structure(.Data=c(0,0,0,0,0,
       0,0,0,0,0,
       0,0,0,0,0),.Dim=c(3,5)),
mb=c(2,-2,-2),
prb=c(3,3,3),
rho=c(0.5,0.5,0.5)
)
```

7. WinBugs code for the weight gain analysis

Model

```
model{

sw[1] <- 0
for (i in 1:Ni) { # LOOP OVER ARMS
       r[i] ~ dbin(p[i],n[i])
       logit(p[i]) <- mu[s[i]]+delta[i]*(1-equals(t[i],b[i]))

       #Random effects model for log-odds ratios
       delta[i] ~ dnorm(md[i],taud[i])
       taud[i] <- tau * (1 + equals(arm[i],3) /3)
       md[i] <- d[t[i]] - d[b[i]] + equals(arm[i],3) * sw[i]

       #Deviance residuals for data i
       rhat[i] <- p[i] * n[i]
       dev[i] <- 2 * (r[i] * (log(r[i])-log(rhat[i])) + (n[i]-r[i]) * (log(n[i]-r[i])
- log(n[i]-rhat[i])))
}

sumdev <- sum(dev[])

#Adjustment for 3 arm trials
for (i in 2:Ni) { sw[i] <- (delta[i-1] - d[t[i-1]] + d[b[i-1]] ) /2 }

#Priors
for (j in 1:Nj) { mu[j] ~ dnorm(0,.0001) }
tau <- 1/(sd*sd)
```

```
sd ~ dunif(0,2)

#Give priors for log-odds ratios
d[1] <-0
for (k in 2:Nk) { d[k] ~ dnorm(0,.0001) }

#All pairwise odds ratios
#or[c,k] is the odds ratio of treatment k relative to treatment c
for (c in 1:(Nk-1)) {
	for (k in (c+1):Nk) {
		or[c,k] <- exp(d[k] - d[c])
	}
}

#Odds and probabilities of weight gain for each treatment
prob[1] <- 33/613 #Baseline estimate for weight gain associated with PLB depot
(treatment 1)
odds[1] <- prob[1] / (1-prob[1])

for (k in 2:Nk) {
	odds[k] <- or[1,k] * prob[1] / (1-prob[1])
	prob[k] <- odds[k] / (1+odds[k])
}

# Code for estimating that each treatment is the best option
for (k in 1:Nk) {
 rk[k] <- rank(d[],k) # assumes events are “bad”
best[k] <- equals(rk[k],1) #calculate probability that treat k is best

}


}
```

Data – Weight gain

```
list(

Nk = 7, # treatments
Nj= 5, # trials
Ni= 12, # arms

r=c(42,43,8,12,6,12,100,68,17,7,52,50),

n=c(264,266,131,206,204,144,599,322,269,134,338,346),

s=c(1,1,1,2,2,3,3,3,4,4,5,5),

t=c(2,6,1,4,1,1,3,7,2,1,5,4),

b=c(1,1,1,1,1,1,1,1,1,1,5,5),

arm=c(1,2,3,1,2,1,2,3,1,2,1,2)

)
```

Data – EPS

```
list(

Nk = 7, # treatments
Nj= 5, # trials
```

```
Ni= 12, # arms

r=c(52,46,18,21,12,12,52,28,45,14,76,67),

n=c(265,266,131,206,204,144,599,322,269,134,370,379),

s=c(1,1,1,2,2,3,3,3,4,4,5,5),

t=c(2,6,1,4,1,1,3,7,2,1,5,4),

b=c(1,1,1,1,1,1,1,1,1,1,5,5),

arm=c(1,2,3,1,2,1,2,3,1,2,1,2)

)
```

Initial values

```
#initial values

list(
d=c(NA,0,0,0,0, 0,0),
sd=1,
mu=c(0,0,0,0,0),
delta=c(0,0,0,0,0, 0,0,0,0,0, 0,0)
)
```
